# *Xenopus laevis* as a Model Organism for the Study of Spinal Cord Formation, Development, Function and Regeneration

**DOI:** 10.3389/fncir.2017.00090

**Published:** 2017-11-23

**Authors:** Laura N. Borodinsky

**Affiliations:** Department of Physiology & Membrane Biology and Institute for Pediatric Regenerative Medicine, Shriners Hospital for Children, University of California Davis School of Medicine, Sacramento, CA, United States

**Keywords:** neural tube defects, spinal cord injury, morphogenetic proteins, sensorimotor response, spinal neuron differentiation, axon guidance, calcium-dependent activity, neural plasticity

## Abstract

The spinal cord is the first central nervous system structure to develop during vertebrate embryogenesis, underscoring its importance to the organism. Because of its early formation, accessibility to the developing spinal cord in amniotes is challenging, often invasive and the experimental approaches amenable to model systems like mammals are limited. In contrast, amphibians, in general and the African-clawed frog *Xenopus laevis*, in particular, offer model systems in which the formation of the spinal cord, the differentiation of spinal neurons and glia and the establishment of spinal neuron and neuromuscular synapses can be easily investigated with minimal perturbations to the whole organism. The significant advances on gene editing and microscopy along with the recent completion of the *Xenopus laevis* genome sequencing have reinvigorated the use of this classic model species to elucidate the mechanisms of spinal cord formation, development, function and regeneration.

## Introduction

The use of model organisms has been crucial to the progress toward understanding spinal cord function. The frog has been a pioneer animal model for the study of spinal cord formation, spinal cell specification and differentiation, spinal neuron axon guidance, neuromuscular junction formation and plasticity as well as spinal cord injury and regeneration.

Among amphibians, *Xenopus laevis* has been an advantageous frog species for the study of the spinal cord throughout development and in adulthood for several reasons. First, the *Xenopus laevis* egg is approximately 1-mm diameter this means that is over 2300 times bigger than the mouse egg, the most popular vertebrate species. This allows for accessible manipulation of gene expression and genetic engineering by simple microinjection of constructs into the egg or fertilized embryo. Second, the eggshell is transparent, enabling the direct visualization of the first stages of spinal cord morphogenesis through non-invasive imaging approaches. Third, microinjections at individual blastomeres of the 2- to 32-cell stage embryos result in mosaic genetic manipulation, which can render internal control or tissue specificity for the targeted gene misexpression. Fourth, the development of *Xenopus laevis* spinal cord occurs much faster than for rodents and the developmental rate can be adjusted by growing animals at different temperatures. Fifth, the organization of the spinal cord is simpler than for higher vertebrates with overall fewer spinal cells, fewer types of spinal neurons and fewer connections between them. Nevertheless, the main kinds of spinal cord cells are represented, i.e., sensory neurons, interneurons, motor neurons and glial cells. Sixth, this species exhibits remarkable regenerative capacity during development, including the repair of the injured spinal cord, allowing for the identification of factors that enable successful spinal cord regeneration that might be missing in higher vertebrates. In spite of the unique characteristics of this species, many fundamental processes at the cellular and molecular level are highly conserved across vertebrates, making *Xenopus laevis* a valuable organism to study the mechanisms of human disease.

The features of *Xenopus laevis* mentioned above are few of the reasons why this species have become a successful model to study the first steps of neural induction and spinal cord morphogenesis, spinal neuron differentiation, synaptogenesis, including neuromuscular junction formation and maturation, as well as synaptic plasticity and regeneration after spinal cord injury. Here we compile both the pioneering and current studies that have made significant contributions at both establishing *Xenopus laevis* as a powerful model to study all aspects of spinal cord development and function and advancing the field of spinal cord research by uncovering the mechanisms underlying its development, plasticity and repair.

## First Step in the Formation of the Spinal Cord: Neural Induction in *Xenopus laevis*

Neural induction in vertebrates is the process by which a subset of ectodermal cells commits to the neural phenotype to originate the brain and spinal cord through interactions of the induced cells with neighboring cell layers (Spemann and Mangold, 1924). This event occurs very early in embryogenesis between fertilization and gastrulation, underscoring the importance of generating the neural tissue early on for the success of a viable organism. The first molecular signatures involved in this process were discovered through studies in *Xenopus laevis*. By assessing spatiotemporal expression of tissue-specific proteins, seminal studies showed that non-neural ectodermal cells express a set of proteins that are missing from neuroectodermal cells (Akers et al., 1986). Conversely, the expression of specific neural proteins like neural cell adhesion molecule (N-CAM), the homeobox transcription factor XiHbox8, are identified a couple of hours after *Xenopus laevis* gastrulation (Jacobson and Rutishauser, 1986; Sharpe et al., 1987), although the mRNA for N-CAM for instance is already detected during late gastrulation, after the mesoderm contacts the ectoderm (Kintner and Melton, 1987), suggesting that expression of neural-specific proteins is a step that occurs after neural induction (Jacobson and Rutishauser, 1986). The identification of the inducing tissues resulted in a model that supports the interaction between the mesoderm and the dorsal ectoderm (Spemann, 1938; Smith and Slack, 1983; Sharpe et al., 1987). These interactions are organized by two centers identified in *Xenopus laevis* and called the Nieuwkoop center, located in dorsal-vegetal cells, which expresses Nodal-related endomesodermal inducers and the blastula Chordin- and Noggin-expressing center located in dorsal animal cells that contains both prospective neuroectoderm and Spemann organizer precursor cells (Kuroda et al., 2004).

The factors secreted by inducing cells that drive the commitment to the neural phenotype include Noggin (Lamb et al., 1993), Chordin (Sasai et al., 1995), Follistatin (Hemmati-Brivanlou et al., 1994), Xnr3 (Hansen et al., 1997), all inhibitors of the Bone Morphogenetic Protein (BMP) pathway and Fibroblast Growth Factor (FGF; Kengaku and Okamoto, 1993). The signaling elicited by these factors in the dorsal ectoderm involves translocation of Protein Kinase C to the membrane (Otte et al., 1988, 1991; Otte and Moon, 1992) and recruitment of signaling molecules like Smad10 (Lesueur and Graff, 1999; LeSueur et al., 2002) and the phosphorylation of Smad1 in the linker domain that inhibits its transcriptional activity and nuclear translocation, serving as an integration of several signaling pathways (Pera et al., 2003).

The two models that have emerged from these founding studies have supported a first model of ectodermal cells becoming neural by default, through inhibiting the BMP pathway; in the second model FGF instructs the dorsal ectodermal cells to become neural. More recently, the merging of these two models was proposed (Marchal et al., 2009). By antagonizing BMP signaling through the expression of a dominant negative (DN) cofactor of the BMP pathway, *Xenopus* epidermis was converted into neural tissue. However, this is prevented when FGF4 is knocked down; moreover, BMP signaling inhibition upregulates FGF4 expression. These findings led to the unified model for neural induction dependent on BMP inhibition that in turn activates FGF, which instructs expression of neural phenotype regulators (Marchal et al., 2009).

The factors that drive the neural phenotype downstream neural induction have also been identified by pioneering research in *Xenopus laevis*, and then findings extended to higher vertebrates. Prominent transcription factors are early downstream drivers of the neural commitment and include Zic3 (Nakata et al., 1997), SoxD (Mizuseki et al., 1998b), Zic-r1 and Sox2 (Mizuseki et al., 1998a), which then directly promote expression of proneural genes initiating neural and neuronal differentiation, or they work synergistically with other pathways to initiate expression of the neural phenotype (Mizuseki et al., 1998a).

More recent studies in this model system have revealed novel players in the process of neural induction and early neural specification. Norrin was identified as required for neuroectoderm specification by recruiting β-catenin and inhibiting BMP/TGF-β, thus coordinating these two major signaling pathways (Xu et al., 2012). Efforts have been made to identify the mechanisms responsible for global shifts in gene expression when ectodermal cells become restricted to the neural identity. A study in *Xenopus laevis* led to the discovery that the activity of histone methyltransferase is crucial for the upregulation in the expression of genes controlling neural induction, likely through the repression of the Oct4-related *Xenopus* gene Oct-25 (Nicetto et al., 2013).

## Spinal Cord Formation Through the Lens of *Xenopus laevis* Neurulation

Once the neuroectoderm is induced the emerging neural plate, which appears as a dorsal thickening of the ectoderm, transits through dramatic morphogenic events to transform itself into the neural tube, the precursor of the spinal cord and the brain. Studies in *Xenopus laevis* have come once again to illuminate onto the cellular and molecular mechanisms that mediate neural tube formation. A combination of biochemical signals and mechanical forces orchestrate changes in cell shape, including apical constriction and cell elongation, necessary for the bending of the neural plate, the elevation of neural folds followed by the lateromedial migration and cell intercalation that leads to the mediolateral narrowing and anteroposterior elongation of the neural tissue, process known as convergent extension (for recent review see Sokol, 2016).

Apicobasal cell elongation accounts for thickening and narrowing of the neuroepithelium. In addition, mediolateral cell intercalation within each layer narrows the neural plate in the transverse axis while elongating it in the anteroposterior axis (Keller et al., 1992a,b,c). Neural plate shaping is closely followed by its bending. Initially, a furrow is formed in the notoplate as a result of the medial hinge point cell apical constriction. Then neural plate lateral edges rise forming incipient neural folds. Subsequent folding includes further neural fold elevation, convergence and rolling towards the midline. Continuing apicobasal elongation, cell apical constriction, crawling of lateral neural plate cells under the epidermis and continuing mediolateral intercalation are involved in this phase. During neural plate folding cell intercalation occurs at a higher rate compared to the shaping phase, accelerating convergent extension of the neuroepithelium (Jacobson et al., 1986). Subsequently, neural fold tips come into contact and fuse at the dorsal midline (Davidson and Keller, 1999). Finally, radial intercalation between the two cell layers forms a single-layered neural tube (Edlund et al., 2013).

As neurulation progresses different populations of cells in the neuroepithelium, it exhibit diverse types of cell behaviors (Wallingford, 2005). Apical constriction of superficial neural plate cells requires a precise spatiotemporal regulation of cytoskeletal and cell adhesion molecule dynamics. It was discovered in *Xenopus* that the actin binding protein Shroom is necessary for neural tube closure in amphibians and mammals by inducing apical constriction through enriching the apical neural plate surface with actin filaments and recruitment of the small GTPase Rap1 (Haigo et al., 2003). Neural plate cells exhibit Ca^2+^ dynamics (Wallingford et al., 2001) which elicit contraction events in these cells driven by transient contractile apical pools of actin (Christodoulou and Skourides, 2015).

Apical constriction also requires endocytosis at the apical neural plate cell membrane (Lee and Harland, 2010). A more recent study showed that through the activity of the Planar Cell Polarity pathway, Rab11-positive recycling endosomes localize to the medial apical cell junctions during neural tube formation; this polarization is necessary for neural plate folding (Ossipova et al., 2014). Moreover, a recently published study from our lab demonstrates that endocytosis of the adherens junction molecule C-cadherin is disrupted when folate receptor-1 is downregulated from the apical membranes of superficial neural plate cells, impairing neural plate cell apical constriction and neural tube formation (Balashova et al., 2017). The study provides a mechanism for folate action during neural tube formation that explains the vulnerability of this process to folate disturbances and the benefits of folate supplementation in the prevention of neural tube defects in humans (Blom et al., 2006). Our study, through the use of *Xenopus laevis* as a model system, uncovers specific functions of folate and its receptor beyond its role as a vitamin and enabler of DNA synthesis, since cell division is not necessary for neural tube formation in *Xenopus* (Harris and Hartenstein, 1991), in spite of the necessity of folate/folate receptor-1 for appropriate neural plate cell apical constriction and neural tube closure in this species (Balashova et al., 2017).

**Figure 1:**
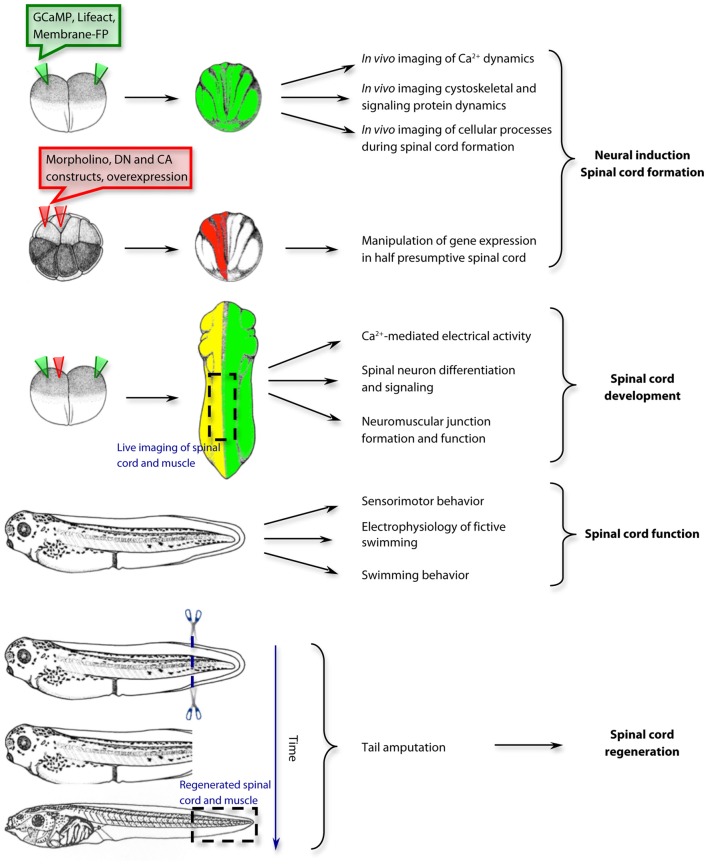
Examples of approaches and applications for the study of spinal cord development, function and regeneration in *Xenopus laevis*. The use of *Xenopus laevis* as a model system spans from the first steps of neural induction and spinal cord formation to the mechanisms of spinal cord regeneration. GCaMP, genetically-encoded Ca^2+^ sensor; Lifeact, F-actin biosensor; Membrane-FP, Fluorescent protein tethered to the plasma membrane; DN, dominant negative; CA, constitutively active. Drawings of embryos and tadpoles were adapted from Xenbase.org and published previously by Nieuwkoop and Faber (1994); Copyright © 1994, Garland Publishing Inc.

The process of convergent extension occurs through cell elongation and intercalation and is also dependent on Ca^2+^ dynamics (Wallingford et al., 2001). In addition, the non-canonical Wnt signaling pathway dependent on Disheveled is necessary for convergent extension (Wallingford and Harland, 2001). Other signaling pathway that has been identified as necessary in regulating microtubule dynamics for *Xenopus* neural fold elevation and cell intercalation is the Repulsive Guidance Molecule-Neogenin interaction (Kee et al., 2008). Microtubule polymerization is also the target of the Zn^2+^ transporter ZIP12 that when knocked down impairs *Xenopus* neural tube formation and neurite extension in developing spinal neurons (Chowanadisai et al., 2013; Figure 1).

## Spinal Neuron Differentiation and Spinal Cord Maturation

The development of spinal cord function relies on the assignment of specific identities to spinal neurons and glia followed by the establishment of synaptic connections. Our current understanding of the functional differentiation of spinal neurons has nourished from studies in *Xenopus laevis*. Pioneering studies by Spitzer and Baccaglini (1976) investigated the development of electrical excitability in developing spinal neurons both *in vivo* and *in vitro*. They found that shortly after neural tube closure Ca^2+^-dependent action potentials are elicited in immature sensory spinal neurons *in vivo* (Spitzer and Baccaglini, 1976). As maturation progresses, the action potential depends on both, Na^+^ and Ca^2+^, and gradually, the Na^+^-driven component of the action potential becomes more predominant, until the Ca^2+^-dependent component disappears, after 3 days postfertilization (Spitzer and Baccaglini, 1976). The shift from the Ca^2+^-dependent to Na^+^-mediated action potential in developing spinal neurons depends on ion channel expression (O’Dowd, 1983; O’Dowd et al., 1988). In particular the upregulation of the delayed-rectifying K^+^ current is crucial to the switch (Ribera and Spitzer, 1989; Burger and Ribera, 1996; Gurantz et al., 1996; Ribera, 1996).

The discovery of the Ca^2+^-dependent action potential in developing neurons opened up a prominent and influential research field focused on the role and the mechanisms of Ca^2+^-mediated electrical activity during nervous system development. In *Xenopus laevis* immature spinal neurons, the mechanisms underlying the spontaneous Ca^2+^ spikes involve the activation of T-type Ca^2+^ channels (Gu and Spitzer, 1993) through a spontaneous initial depolarization which appear to be facilitated by neurotransmitter signaling (Root et al., 2008). This activity is important for the differentiation and maturation of spinal neurons (Gu and Spitzer, 1995). In particular, neurotransmitter phenotype specification is dependent on the level of Ca^2+^ spike activity, with higher frequencies driving the expression of inhibitory neurotransmitter phenotypes and lower frequencies driving expression of excitatory neurotransmitter phenotypes (Borodinsky et al., 2004; Marek et al., 2010). The changes in neurotransmitter phenotype are accompanied by changes in neurotransmitter receptor in target cells including the skeletal muscle in tadpole’s neuromuscular junctions (Borodinsky and Spitzer, 2007; Figure 1).

We learned from studies on the patterning of ventral and dorsal spinal neuron identities in *Xenopus laevis*, the dependence of this process on the notochord; notochordless embryos exhibit fewer number of motor neurons and higher number of sensory neurons, and commissural interneuron axons cross the midline in a disorganized manner overall resulting in perturbed left-right alternation of the locomotive behavior (Clarke et al., 1991). Moreover, studies in our lab have demonstrated that the Ca^2+^-mediated electrical activity interacts with other essential developmental signaling pathways like Sonic hedgehog (Shh) and BMPs to orchestrate the differentiation of spinal neurons (Belgacem and Borodinsky, 2011, 2015; Swapna and Borodinsky, 2012; Borodinsky et al., 2015). Shh enhances activity of spinal neurons (Belgacem and Borodinsky, 2011) while BMP inhibits it (Swapna and Borodinsky, 2012). These studies identified novel non-canonical signaling pathways for morphogenetic protein action in the spinal cord. Furthermore, we discovered that Ca^2+^ spike activity is the mechanism by which Shh canonical signaling switches off during the early stages of spinal cord development (Belgacem and Borodinsky, 2015), phenomenon that is apparent in frogs and mice (Lee et al., 1997; Balaskas et al., 2012).

This model system has also been crucial in the identification of the mechanisms governing axon guidance of developing spinal neurons. Studies from the Poo laboratory first used *Xenopus* spinal neurons grown in culture to identify the signaling mechanisms underlying chemoattraction and repulsion of extending growth cones. They found that localized changes in levels of cyclic nucleotides transduce the signal of guidance cues like Netrin to change the direction of the growing axon (Lohof et al., 1992; Zheng et al., 1994; Ming et al., 1997, 1999, 2001; Song et al., 1997, 1998; Xiang et al., 2002). Further studies demonstrated that these mechanisms occur *in vivo* and the signaling is also dependent on localized Ca^2+^ dynamics in growth cones and filopodia (Gomez et al., 2001; Ming et al., 2001; Robles et al., 2003; Henley et al., 2004; Shim et al., 2005; Wang and Poo, 2005; Robles and Gomez, 2006).

In addition to the extension and direction of axon growth, *Xenopus laevis* spinal neurons have been used to determine the mechanisms underlying neuronal polarity through the establishement of dendrites vs. axons. *Xenopus* commissural spinal interneurons exposed to semaphorin 3A revert the identity of axons to dendrites by activating Ca_v_2.3 channels through the cGMP-mediated activation of PKG (Nishiyama et al., 2011).

## Advances in the Understanding of Spinal Cord Function Through Research in *Xenopus*

The spinal cord is in charge of sensorimotor functions in vertebrates. The elucidation of the mechanisms involved in the establishment of the sensorimotor function during development and the identification of the circuitry responsible for eliciting a specific sensorimotor response have been common foci of research. *Xenopus* offers an excellent system to study these aspects of spinal cord development and function. In particular, two important fronts of research have benefited greatly from the studies done in *Xenopus*; one is the research on the mechanisms of neuromuscular junction plasticity during development, and the other is the identification of the circuitries in the spinal cord that elicit the simple sensorimotor reflexes and the more complex locomotor behaviors (Figure 1).

Taking advantage of the ease of *in vivo* time-lapse imaging in developing tadpoles, the Cline lab fluorescently labeled spinal motor neuron axons and nicotinic receptor clusters in muscle cells through electroporation, and demonstrated that axon branches and synaptogenesis are concurrent and dynamic (Javaherian and Cline, 2005). They further identified the Candidate Plasticity Gene 15 as necessary for axon arborization and promotion of synaptogenesis at the neuromuscular junction (Javaherian and Cline, 2005). The rules of pre and postsynaptic interactions that govern plasticity events such as synaptic depression and potentiation were first tested in *Xenopus laevis* neuron-muscle connections, a simple prototype of developing vertebrate neuromuscular junction. A transient increase in intracellular Ca^2+^ concentration in the connected muscle cell triggers a decrease in neurotransmitter release from the presynaptic motor neuron. This depression is persistent and dependent on the connection with the affected muscle cell and not on a secreted extracellular factor (Cash et al., 1996). In contrast, a burst of action potentials in the presynaptic motor neuron leads to a sustained potentiation of synaptic activity of the neuromuscular junction due to increase in probability of neurotransmitter release (Wan and Poo, 1999). Neurotrophins were identified in *Xenopus* neuromuscular junction studies as effective mediators of synaptic potentiation. In particular Brain-Derived Neurotrophic Factor (BDNF) was found to potentiate synaptic efficacy, through cAMP signaling (Boulanger and Poo, 1999a), when paired with a presynaptic depolarization of the motor neuron within a specific timing (Boulanger and Poo, 1999b). A more recent study has further the understanding of BDNF action on activity-dependent neuromuscular junction remodeling by demonstrating that stimulating motor neurons induces the maturation of BDNF in the presynapse. While immature BDNF promotes retraction of the less active synaptic terminal, mature BDNF leads to stabilization of the active terminal (Je et al., 2012).

With regard to the understanding of the circuitry underlying locomotor behavior in vertebrates, seminal work from Roberts et al. (1981) took advantage of the simplicity of the neuroanatomy and behavior of swimming larvae to answer fundamental questions on the connectivity and organization of the underlying neuronal networks. They discovered that the rhythmicity of the swimming behavior resides in the spinal cord and is inherent to each side of the cord (Roberts et al., 1981). They also found that the commissural inhibitory spinal interneurons are necessary for the strict alternation between left and right motor neuron activity (Roberts et al., 1981). Other studies have been equally insightful at revealing the circuitry underlying the sensorimotor response upon touch and the mechanisms responsible for the relationship between the locomotor central pattern generator and motor neuron activity (Sillar and Roberts, 1988; Li et al., 2003; Buhl et al., 2015).

The neurophysiological substrate for the acquisition of progressive refinement in the locomotor system has been investigated by Sillar and Roberts (1988). They found that changes in motor neuron firing properties and patterns of innervation of the axial musculature in *Xenopus laevis* larvae change during the first day after hatching so that different pools of motor neurons progressively innervate a more restricted region of muscle fibers (Zhang et al., 2011). The motor neuron firing probability transitions from a single action potential per swim cycle right after hatching to different firing probabilities 1 day after hatching, when diverse types of swimming behavior are elicited (Zhang et al., 2011).

## Spinal Cord Regeneration

All the aspects of spinal cord development and function discussed above highlight the usefulness of *Xenopus* as a model system because the processes occurring in the frog are conserved across vertebrates including mammals, with the advantage of being a simpler system, more amenable to *in vivo* and *in vitro* approaches, live-imaging and physiology studies. Thus, this model system enables the identification of the molecular and cellular mechanisms underlying the conserved and fundamental processes. Other features of *Xenopus* make this animal model unique among other systems. This uniqueness can also be advantageous; for instance, from investigating the mechanisms that make this system different than other vertebrates we can better understand the potential limitations that other species including humans face. Before metamorphosis, the regenerative capacity of frogs, and *Xenopus* in particular, is remarkable. Even the adult frog is able to regenerate certain axons like the optic nerve but not the spinal axons, due to some non-permissive factors generated by the spinal cord oligodendrocytes and myelin (Lang et al., 1995). Outstandingly, when the tadpole’s tail is amputated, all the tissues including skin, notochord, muscle and spinal cord regenerate (Figure 1). Every tissue regenerates from a dedicated pool of stem cells that are activated upon amputation (Gargioli and Slack, 2004). Interestingly, the *Xenopus* tadpole tail exhibits this regenerative capacity throughout development with the exception of a so-called refractory period when the tail heals without regeneration. This further uniqueness enables the study within the same model system of the factors that are permissive of the regenerative process. The Slack laboratory took advantage of this aspect of *Xenopus laevis* and discovered that BMP and Notch signaling (downstream of BMP) are necessary for tail regeneration and sufficient for overriding the refractory period (Beck et al., 2003). BMP signaling appears to be required for appropriate neural cell proliferation to replenish the spinal cord (Beck et al., 2006). It has also been shown that a restricted rate of apoptosis is required during the first 24 h post injury for successful regeneration during the permissive stages and that a more extensive apoptosis may interfere with appropriate regeneration during the refractory stages (Tseng et al., 2007). The extent of apoptosis appears to be controlled by the activity of the V-ATPase H^+^ pump that is upregulated in the regenerating tissue and repolarizes the membrane potential of regenerating cells after an initial depolarization is triggered by the injury (Adams et al., 2007). In agreement with these studies we found that cells from the regenerating tail exhibit spontaneous Ca^2+^ transients during the first 24 h post amputation. Suppressing this activity impairs tail regeneration, apparently by impairing activation of stem cells for replenishing the spinal cord and muscle (Tu and Borodinsky, 2014). In recent studies gene expression profiles during tail regeneration were collected from *Xenopus laevis* (Lee-Liu et al., 2014) and *tropicalis* (Love et al., 2011) amputated tadpoles at different periods post injury and in regenerative and non-regenerative stages. From these excellent resources, insightful studies have emerged demonstrating that injury increases production of reactive oxygen species that are upstream Wnt/β-catenin and the upregulation of FGF20 expression, which contribute to the signaling mechanisms necessary for tail regeneration (Love et al., 2013).

## Concluding Remarks

*Xenopus* is a classic model for embryology and physiology research and has been perfectly suited for studies on the formation, development, maturation and repair of the spinal cord. The recent completion of the sequencing of the *Xenopus laevis* genome (Session et al., 2016), and *Xenopus* *tropicalis* genome since 2010 (Hellsten et al., 2010), allows for the use of both in genetic studies; along with the remarkable advances in systems biology, including genomics, metabolomics and proteomics, the opportunities for discovery that this model system offers are reinvigorated (Lombard-Banek et al., 2017; Tandon et al., 2017). Additionally the accessibility of novel approaches for gene editing like TALENs (transcription activator-like effector nucleases) and CRISPR-Cas (clustered regularly interspaced short palindromic repeats-CRISPR associated nucleases) expand the use of *Xenopus* as a genetically tractable model for loss and gain of function studies (Tandon et al., 2017). Salient questions remain in every aspect of spinal cord research that was covered in this review and other aspects that were not included. The signaling mechanisms affecting the formation of the spinal cord and responsible for birth defects like spina bifida demand further investigation (Wallingford et al., 2013). The deeper understanding of the mechanisms governing the switch from neural stem cell to neuron is important for both the prevention of spinal cord malformations during development and the promotion of the recovery and regeneration in patients with spinal cord injury. Finally, studies on the assembly and plasticity of circuitry underlying spinal cord function can benefit from the advances in microscopy and optogenetics to dissect out the precise interactions among different types of neurons. The prediction is that *Xenopus* will continue to offer an advantageous platform for hypothesis-driven research that will contribute to the understanding of spinal cord development, function and disease.

## Author Contributions

LNB wrote the manuscript.

## Conflict of Interest Statement

The author declares that the research was conducted in the absence of any commercial or financial relationships that could be construed as a potential conflict of interest.
